# Targeting Glutaminolysis: New Perspectives to Understand Cancer Development and Novel Strategies for Potential Target Therapies

**DOI:** 10.3389/fonc.2020.589508

**Published:** 2020-10-26

**Authors:** Zhefang Wang, Fanyu Liu, Ningbo Fan, Chenghui Zhou, Dai Li, Thomas Macvicar, Qiongzhu Dong, Christiane J. Bruns, Yue Zhao

**Affiliations:** ^1^ Department of General, Visceral, Tumor and Transplantation Surgery, University Hospital Cologne, Cologne, Germany; ^2^ Interfaculty Institute for Cell Biology, University of Tübingen, Tübingen, Germany; ^3^ Department of Anesthesiology, Changhai Hospital, Naval Medical University, Shanghai, China; ^4^ Max-Planck-Institute for Biology of Ageing, Cologne, Germany; ^5^ Department of General Surgery, Huashan Hospital & Cancer Metastasis Institute & Institutes of Biomedical Sciences, Fudan University, Shanghai, China

**Keywords:** glutaminolysis, cancer metabolism, metastasis, glutaminase inhibitor, combination therapy

## Abstract

Metabolism rewiring is an important hallmark of cancers. Being one of the most abundant free amino acids in the human blood, glutamine supports bioenergetics and biosynthesis, tumor growth, and the production of antioxidants through glutaminolysis in cancers. In glutamine dependent cancer cells, more than half of the tricarboxylic/critic acid (TCA) metabolites are derived from glutamine. Glutaminolysis controls the process of converting glutamine into TCA cycle metabolites through the regulation of multiple enzymes, among which the glutaminase shows the importance as the very first step in this process. Targeting glutaminolysis *via* glutaminase inhibition emerges as a promising strategy to disrupt cancer metabolism and tumor progression. Here, we review the regulation of glutaminase and the role of glutaminase in cancer metabolism and metastasis. Furthermore, we highlight the glutaminase inhibitor based metabolic therapy strategy and their potential applications in clinical scenarios.

## Introduction

Sustained and unhindered proliferative tumor cells require high levels of energy and building block molecules which depend partially on the availability of nutrients and oxygen in the microenvironment. In 2011, Hanahan et al. described reprogramming of energy metabolism as an emerging hallmark of neoplastic disease (1). Pavlova et al. further summarized six cancer associated metabolic changes, including enormous influx of nutrients, re-shaped nutrient acquisition under hostile condition, use of intermediates of glycolysis/citric acid cycle (TCA cycle) for biosynthesis and NADPH production, increased nitrogen demand, altered epigenetic modification of metabolism related genes together with changes in post-transcriptional modification (PTM) of the enzymes, and ultimately the metabolic interaction with the microenvironment (2).

Reprogrammed metabolism characterized by the markedly increased consumption of glucose and glutamine is emphasized when several examples have revealed that it could support tumor cell survival and biosynthesis (3, 4). Not merely an increased glucose uptake, remodeled glucose catabolic pattern is also considered as a feature of proliferating cells. Cancer cells preferentially utilize glucose in an oxygen “independent” way, in which they convert most pyruvate to lactate rather than delivering them into TCA cycle for a higher ATP yield (described as ‘Warburg effect’) (5). A widely accepted theory rationalizing such phenomenon is that aerobic glycolysis provides abundant intermediates for a quick *de novo* synthesis of nucleotides, non-essential amino acids (NEAAs) and fatty acids and certainly, a more rapid ATP supplementation than TCA cycle (6, 7).

Glutamine is the most abundant amino acid in blood and muscle, which provides a stable nitrogen and carbon pool for protein, nucleotide, and lipid biosynthesis (8). After first evidenced by Eagle et al. that the glutamine consumption in HeLa cells is 10 to 100 times higher than any other amino acids (9), augmented glutamine metabolism has been reported to be significantly linked with tumor growth, invasion, and metastasis in various cancer types (e.g. ovarian cancer, breast cancer, and pancreatic cancer) (2, 10). Due to the diversion of pyruvate from entering TCA cycle, cancer cells rely more on glutamine carbon for anaplerosis (10, 11). Given the crucial role of glutamine in bioenergetics and biosynthesis in cancers, the study on glutamine metabolism could ensure a better understanding of cancer progression, thus further inspiring the development of potential methods of targeted therapy. In this review, we focus on the current understanding of glutaminase-related glutaminolysis in cancer metabolism. The role of glutaminase in tumorigenesis and their regulation in metastasis are also discussed. Furthermore, the glutaminase inhibitor based metabolic targeted therapies are summarized and highlighted.

## Glutamine Metabolism in Cancer

Glutamine was believed to be a non-essential amino acid in normal physiological condition until 1990, when Lacey et al. firstly uncovered that the supply of glutamine under a catabolic stressed condition failed to meet the demand of this nutrient. Since then, glutamine has been regarded as a conditional essential amino acid (12). Cancer cells undergo aerobic glycolysis (Warburg effect), resulting in restricting pyruvate entry into the TCA cycle. A process known as glutaminolysis replenishes TCA cycle with intermediates from glutamine (13). Using isotopic tracers, a number of studies, including both *in vitro* and *in vivo*, have demonstrated the massive contribution of glutamine to TCA metabolites pool in glutamine dependent cancer cells (11, 14–16). Glutamine-driven oxidative phosphorylation has also been discovered as a major ATP source in transformed mammalian cells (17).

Rapidly-dividing cells including those in kidney, gastrointestinal tract, immune compartments and cancer cells, possess a tremendous appetite for glutamine. For example, deprivation of glutamine induces necrosis of intestinal mucosa and apoptosis in human cell lines (12, 18), while additional oral supplementation of glutamine among cancer patients undergoing radio- and chemotherapy improves mucosa healing and ameliorate life quality (19). Flux of glutamine is mediated by the transporter SLC1A5 (ASCT2) and antiporter SCL7A5/SCL3A2 on cell membrane, and the newly identified SLC1A5 variant on the inner mitochondrial membrane (20, 21). Glutaminolysis in mitochondria starts from the conversion of glutamine to glutamate by glutaminase. Then glutamate metabolism forks into two different ways: either converted by glutamate dehydrogenase (GLUD) into α-KG to fuel TCA cycle, or to join a biosynthetic pathway for the production of NEAAs *via* aminotransferases (e.g. alanine, aspartate, and phosphoserine) (10). Apart from its contribution in bioenergetic and biosynthetic process, glutaminolysis is also directly involved in the regulation of redox homeostasis through the synthesis of glutathione (GSH) by providing glutamate (22). In addition, glutamine could also function as a signaling molecule, such as in the regulation of mTOR pathway (23, 24). Despite the diverse constitution and activity of enzymes involved in glutaminolysis under different cellular status, the maintenance of a sufficient intracellular concentration of glutamate relies predominantly on the activity of phosphate-dependent glutaminase (GLS), whose disrupted expression has been observed in various cancer cell lines (25). Human GLS could roughly be summarized as two isoforms which derive from two different but related genes. The kidney-type (GLS1 or KGA) is ubiquitously expressed in various normal tissues, while the liver-type (GLS2 or LGA) is restricted in the liver, brain and pancreas (26, 27). Unlike the coherent expression tendency of GLS1 in various cancer types (26), the function pattern of GLS2 seems to be more complex and controversial (28, 29). Accumulating evidence has confirmed that the activity of both GLS1 and GLS2 rest highly on the metabolic state of the cells, as GLS1 is activated by high level of phosphate and inhibited by the enzymatic product glutamate, while GLS2 is activated by lower level of phosphate as well as ammonia (30, 31). Though glutaminases are mainly reported as mitochondrial proteins, the localization of KGA in cytosol and GLS2 in nuclei have also been revealed (32, 33). Further discussion of the glutaminase isoenzymes are displayed in the following parts.

Besides, as one key component in cellular intermediary metabolism, glutamine can act either as nitrogen donor (α- and γ- nitrogen) or carbon donor. While the carbon skeleton from glutamine could directly reserve as a carbon reservoir in protein and fatty acid synthesis, the release of a free amide (γ-nitrogen) group exploits its new role in *de novo* biosynthesis for purines and pyrimidines with 2 glutamine derived nitrogen molecules for the purine ring and one nitrogen for pyrimidine ring (34). In addition, glutamine metabolites account partially for the synthesis of fatty acid synthesis in cancer cells with impaired TCA cycle products, e.g. citrate that could support the synthetic process. Such mechanism is mediated by a process called reductive carboxylation, which is briefly described as the conversion of α-ketoglutarate (α-KG) to citrate catalyzed by isocitrate dehydrogenases (IDHs) (35). Studies have observed reductive carboxylation in hypoxic cancer cells *in vitro* and confirmed its importance in supporting lipid genesis for tumor progression *in vivo* (36, 37). Additionally, glutamine metabolites participate in keeping cellular and organismal homeostasis. Free ammonia, which could be released from glutamine catabolism, is a key component for acid-base homeostasis in kidney (38). Enhanced reductive formation of citrate from glutamine by IDHs also supports redox homeostasis and mitigates oxidative oxygen species (ROS), thus cooperatively facilitating spheroid forming in 2 lung cancer cell lines (39). Taken together, altered glutamine metabolism in cancer cells strongly supports tumor growth and progression, which in turn could encourage the investigation for metabolic targeted therapy of cancers.

## Regulation of Glutaminases in Cancer

**Figure 1:**
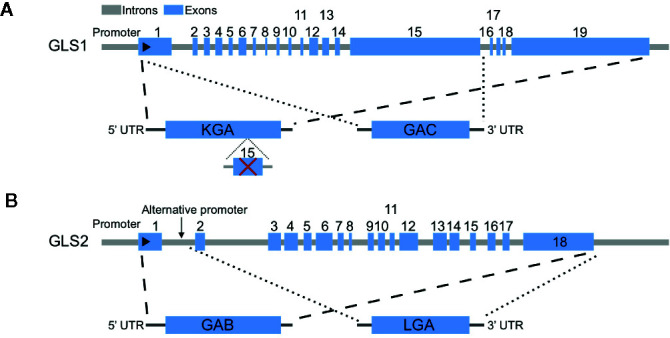
Genomic structures of human GLS1 and GLS2 and alternative transcripts. **(A)** Two alternative transcripts arise from GLS1, KGA, and glutaminase C (GAC). KGA is the longer isoform with all exons except exon 15, while GAC is the shorter isoform with exons 1–15. **(B)** Two alternative transcripts arise from GLS2, GAB, and LGA. GAB is the longer isoform with all exons, while LGA is the shorter isoform lack of exon 1.

Glutaminases are encoded by two different genes called GLS1 and GLS2, and both have longer and shorter isoforms as a result of alternative splicing: KGA and glutaminase C (GAC) for GLS1, and LGA and glutaminase B (GAB) for GLS2 (31) (**Figure 1**). While GLS1 is usually upregulated in cancers, the expression of GLS2 is generally repressed in cancers (26). GAC has higher activities and is the predominant GLS1 isoform in cancers (40–42). Recently, Redis et al. revealed that the alternative splicing of GLS1 is regulated by a long non-coding RNA (lncRNA) called CCAT2, which interacts with CFIm complex and results in the preferential expression of GAC (43). Here, we summarize the key regulators of glutamine metabolism in cancers, focusing on the regulation of glutaminases by oncogenes (c-Myc, KRAS), tumor suppressor (TP53) and other factors.

The oncogene c-Myc has been reported to regulate the expression of several genes in glutamine metabolism, including GLS1 (44), glutamine synthetase (GLUL) (45), GLUD and aminotransferases (46). c-Myc promotes the uptake of glutamine by directly binding to the promoter region of glutamine transporters SLC1A5 and SLC38A5 (47, 48). However, for the regulation of GLS1, c-Myc indirectly promotes the expression of GLS1 through transcriptional repression of miR-23a and miR-23b (44), which are also repressed by NF-κB (49). MYC could also upregulate GLS1 by repressing the expression of an antisense lncRNA GLS-AS (50). Another oncogenic transcriptional factor c-JUN also regulates the gene expression of GLS1 (51). Several pathways regulate the expression of GLS1 through c-MYC have also been reported. The GSK3α/β pathway indirectly upregulates GLS1 through modulating the protein stability of c-Myc and c-Jun (52). The mTORC1/S6K1 pathway positively regulates GLS1 through the eIF4B-dependent control of c-Myc translation (53).

RAS proteins are frequently mutated in many types of human cancers (54). KRAS is the most frequently mutated isoform, especially in pancreatic cancer with more than 90% of the patients (55). Both c-Myc and KRAS have been reported to enhance glycolysis and glutamine addiction, while diverting glucose away from TCA cycle (11, 47). However, the mechanism of glutamine-dependent tumor growth is largely unknown. Son et al. reported a non-canonical pathway of glutamine use in pancreatic ductal adenocarcinoma (PDAC) cells, in which the anabolic metabolism of glutamine is mainly through the glutamic-oxaloacetic transaminase 1 (GOT1) dependent pathway (56). This non-canonical glutamine metabolism also contributes to the maintenance of redox homeostasis in PDAC, and the inhibition of anabolic glutamine metabolism sensitizing PDAC to oxidative stress. Interestingly, it was suggested that different KRAS mutations may show different effects. For instance, KRAS G12V mutation is less glutamine-dependent than G12C or G12D mutation in lung cancer cells (57). However, this difference of glutamine-dependence is not explained by the differential expression of glutaminolysis related enzymes. In addition to the regulation of GOT1 by KRAS, oncogenic PIK3CA mutations also have been reported to mediate metabolic reprograming of glutamine in colorectal cancer (CRC) by upregulating glutamate pyruvate transaminase 2 (GPT2) (58). However, KRAS mutants did not show differential response to glutamine deprivation in case of CRC cell lines. Moreover, NRF2 (nuclear factor erythroid 2-related factor 2) pathway plays a critical role in the metabolic reprogramming to glutamine dependence in KRAS-mutated cells (59, 60). Mukhopadhyay et al. reported that glutamine metabolism was rewired by NRF2, which also promotes chemotherapy resistance in KRAS-driven PDAC cells (59). Galan-Cobo et al. reported that LKB1 (liver kinase B1) and the KEAP1/NRF2 pathways cooperatively drove metabolic reprogramming and enhanced sensitivity to the glutaminase inhibitor CB-839 both *in vitro* and *in vivo* (60).

Hypoxia-inducible factor (HIF) drives metabolic adaptation to hypoxic conditions in many solid tumors (61, 62). Under hypoxic conditions, cells use glutamine to generate citrate by enforcing a shift from glutamine oxidative metabolism towards reductive carboxylation to support proliferation through lipids synthesis (35, 36, 63). Thus, hypoxia is an inducer of reductive metabolism of glutamine in cancers. Furthermore, hypoxia upregulates GLS1 expression in a manner of transcriptional activation by HIF-1α (64).

Besides the transcriptional and post-transcriptional regulation of glutaminase, PTM is also important for the activity of glutaminase (65–67). Wang et al. found that hyperactivation of Rho-GDPase/NF-κB significantly enhanced glutaminase activity by promoting its phosphorylation, while not affecting the expression levels of the enzyme (65). Later on, Han et al. revealed that the key Ser314 phosphorylation site on GAC was regulated by NF-κB-PKCϵ axis (68). In addition, HGF-MET axis is reported to activate GLS activity by phosphorylation, though the phosphorylated site is not indicated (69). Furthermore, mitochondrial desuccinylase SIRT5 stabilizes GLS through desuccinylation of residue K164, which protects GLS from ubiquitin mediated degradation (70).

**Figure 2:**
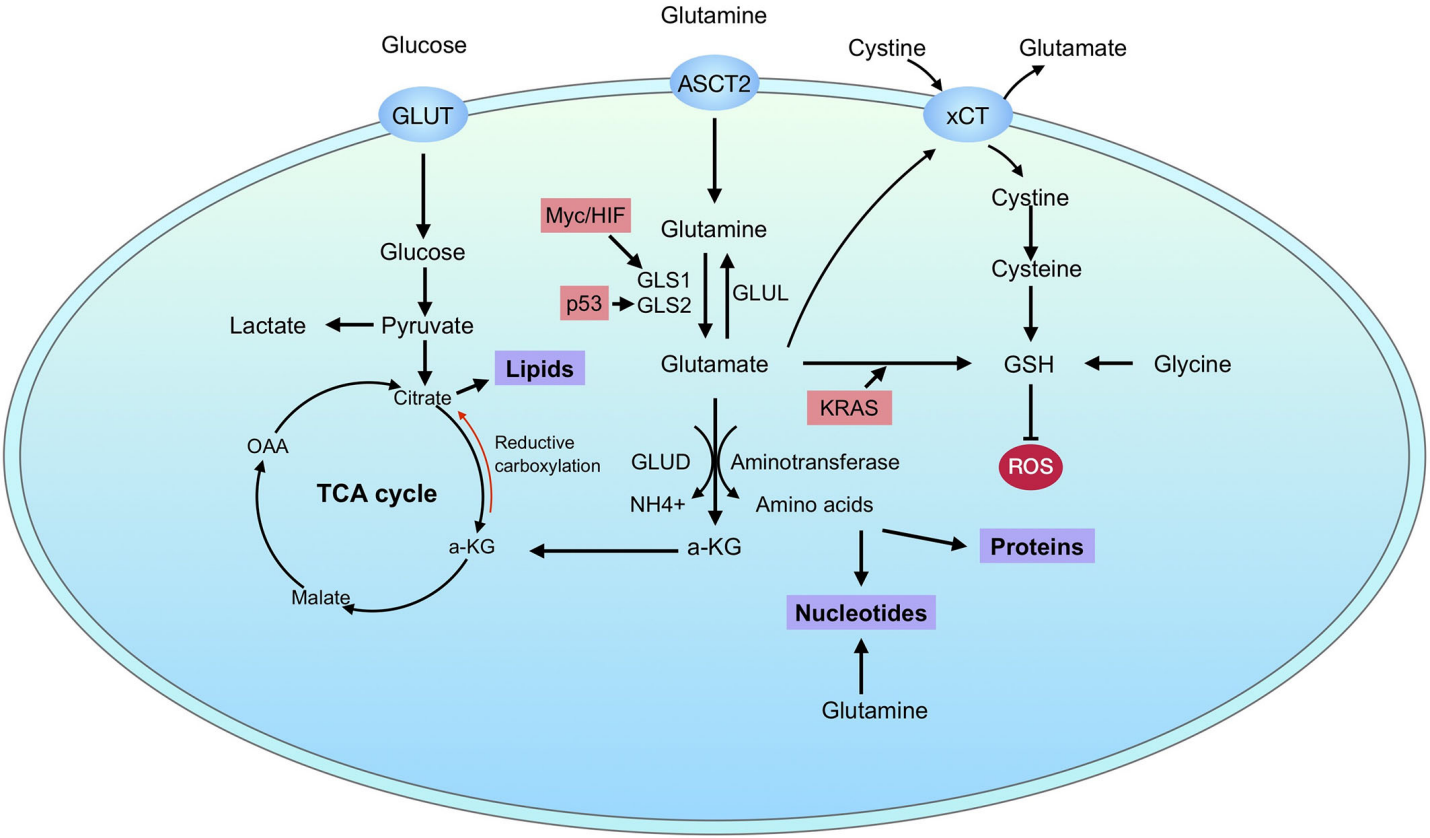
Glutamine metabolism in cancer. Cancer cells uptake glucose and glutamine through GLUT and ASCT2, respectively. After transporting into cells, glutamine is catalyzed to glutamate by glutaminases, which have two isoforms: GLS1 and GLS2. Glutamate is further converted to α-KG through GLUD or aminotransferases. The resulting metabolites can supply for bioenergetics through tricarboxylic/critic acid (TCA) cycle and support biosynthesis of proteins, nucleotides and lipids. In addition, glutamine metabolism also contributes directly to GSH synthesis. The regulation of glutaminase is marked in pink.

GLS2 seems to be regulated in a different way from GLS1’s. GLS2 has been proved to be a target of p53 (71, 72). Interestingly, the regulation of GLS2 by p53 was involved in the regulation of ferroptosis (73, 74). Besides p53, TAp63 and TAp73 as well regulate the expression of GLS2 (75, 76). Differently to GLS1, GLS2 is directly upregulated by N-Myc in neuroblastoma (77). In breast cancer, GLS2 expression is preferentially upregulated in luminal-subtype cancers *via* promoter methylation and GATA3, a master regulator of luminal differentiation (78). Recently, the post translational modification of GLS2 by GCN5L1 has also been revealed, which modulates the oligomerization and acetylation of GLS2 (79). In summary, these observations renew our understanding of glutamine metabolic reprogramming in cancers and contribute to the optimization of glutamine targeting therapy. A summary of the regulation of glutamine metabolism in cancers is depicted in **Figure 2**.

## Opposite Roles of GLS1 and GLS2 in Tumorigenesis

Glutaminase is dysregulated in many cancers, which makes it an appealing target for cancer therapies (22). However, whether the functions of glutaminase is tumorigenic or tumor suppressive remains controversial, especially from the view of isoenzymes (26). Generally, the upregulation of GLS1 links with augmented tumorigenesis, while the expression of GLS2 is more likely related with quiescent or differentiated cell states.

GLS1, a mitochondrial enzyme, hydrolyzes glutamine into glutamate and fuels rapid proliferation of cancer cells. GLS1 might be emphasized as a multiple player in tumorigenesis and progression of human cancers (44, 80). Increased GLS1 expression in a variety of human cancer types was associated with significantly decreased patient survival, which suggests its function as a potential prognostic biomarker for many human cancers, including hepatocellular carcinoma (HCC), ovarian cancer, osteosarcoma, colorectal cancer (CRC) and breast cancer (64, 81–85). Directly or indirectly elevated expression of GLS1 correlates with poor prognosis in these human cancers and GLS1 could be developed as a diagnostic and therapeutic target for these types of cancers (26). Xiang et al. demonstrated that GLS1 expression was required for hypoxia-induced migration and invasion *in vitro* and for tumor growth and metastatic colonization *in vivo* in CRC cells (64). The important role of GLS1 also shows that the overexpression of GLS1 induced metastasis and invasion and promoted epithelial-mesenchymal transition (EMT) in intrahepatic cholangiocarcinoma (ICC) cells (86). In addition, Li et al. demonstrated that targeting GLS1 not only reduced the expression of stemness-related genes including NANOG, OCT4, KLF4, SOX2 and c-Myc, but also suppressed CSC properties *via* ROS/Wnt/β-catenin signaling (81).

Compared with GLS1, GLS2 is more regarded as a tumor suppressor. GLS2 is repressed in glioblastoma, HCC and colon cancers (87–89), while overexpressed in luminal subtype of breast cancer (78). As a target gene of p53, GLS2 shows antioxidant function through regulation of ROS level and GSH/GSSG ratio in cells, contributing to its role in tumor suppression (71, 72, 90). The upregulation of GLS2 in cancer cells induced an antiproliferative response with cell cycle arrested at the G2/M phase and reduced tumor cell colony formation in HCC (33, 71). The researches revealed that GLS2 negatively regulates the PI3K/AKT signaling and plays an important role in tumor suppression in HCC (89). Furthermore, Kuo et al. demonstrated that expression of GLS2 inversely correlates with poor prognosis and early recurrence in HCC patients (91). On the contrary, Dias et al. revealed that GLS2 amplification or overexpression is linked to worse overall, disease-free and distant metastasis-free survival in breast cancer (92). The increased expression of GLS2 leads to enhanced cell migration, invasion and lung metastasis (92). Consistently, Lukey et al. found that the expression of GLS2 supports proliferation and tumorigenesis in luminal subtype breast cancers (78). These data established an unforeseen tumorigenic role of GLS2 in breast cancer.

Interestingly, Ishak Gabra et al. reported that dietary glutamine supplementation inhibited melanoma tumor growth by suppressing epigenetically activated oncogenic pathways (93). The inhibitory effect of glutamine in tumor growth observed here is due to the elevated intra-tumoral α-KG level, consistent with the reported role of α-KG as a tumor suppressor (94, 95). Taken together, GLS1 is more likely to be tumorigenic and a promising therapeutic target, whereas GLS2 behaves more like a tumor suppressor factor despite some controversial results.

## Glutaminolysis and Cancer Metastasis: EMT, Tumor Immunology, and Tumor Microenvironment

In addition to the multiple functions of glutamine metabolism in regulating tumor biology described above, a number of studies have also suggested that glutamine metabolism participated in several aspects of tumor metastasis. By analyzing eight ovarian cancer cell lines, Yang et al. suggested that glutamine dependent ovarian cancer cells showed stronger invasion ability and were related to worse patient survival when compared with glutamine non-dependent cancer cells (83). In addition, suppressing glutamine uptake by inhibiting glutamine transporter ASCT2 significantly inhibited prostate cancer growth and metastasis (96). Moreover, in patient-derived organoids model, Braun et al. found that glutamine was increased more than four times from early-recurrent PDAC patients with the development of tumor recurrence within the first six months after radical surgery, than those from late-recurrent patients, suggesting that glutamine metabolism may be diverse according to different tumor malignancies (97). To date, the exact mechanisms linking glutamine metabolism to tumor metastasis are still unclear, but studies have demonstrated that glutamine may participate in the metastatic process through the interaction with EMT, tumor immunology and tumor microenvironment.

EMT is an important cellular program that enables epithelial cells to acquire a mesenchymal phenotype with increased motility as well as invasive ability, and is widely considered as a critical process for the initiation of the metastatic cascade (98). Glutamine metabolism was reported to be related to EMT in several types of malignant tumors. Takaoka et al. found an inverse correlation between GLS1 and E-cadherin expression by analyzing seven CRC cell lines. And the knockdown of GLS1 not only elevated E-cadherin expression but also suppressed Vimentin and Slug expression in CRC cells, referring to as an EMT induction by GLS1 (99). By transactivating GLS1 and GOT2 to enhance asparagine synthesis, SOX12 overexpression promotes CRC cell proliferation, migration, invasion, and metastasis (100). GLS1 was also reported to promote cell migration and invasion by regulating EMT in intrahepatic cholangiocarcinoma, in which GLS1 expression was higher in tumor tissues than in peritumoral tissues, and the higher expression of GLS1 independently predicted a poor survival (86). Besides, Ramirez-Peña et al. found that experimentally induced EMT breast cancer cells showed a decreased GLS2 expression, which could be further restored by inhibiting the EMT transcription factor FOXC2 (101). GLS2 was found to be capable of repressing cell migration, invasion, and metastasis of HCC through the suppression of EMT secondary to the downregulation of Snail *via* Dicer-miR-34a-Snail axis *in vitro* and *in vivo* (91), suggesting a negative regulation role of GLS2 on EMT. Likewise, GLS2 but not GLS1 could not only inhibit HCC cell migration and invasion *in vitro*, but also suppress lung metastasis in a mouse model through inhibiting Rac1 activity and mediating p53’s function (28). Conversely, Dias et al. reported that GLS2 expression was able to increase the EMT markers as well as cancer cell migration and invasion partly through the regulation of ERK and ZEB1 in breast cancer (92), indicating a positive induction of EMT by GLS2. In general, GLS1 shows a positive regulation of EMT process while the functions of GLS2 on EMT are diverse and may be attributed to different tumor types as well as varying degrees of tumor malignancy.

Immune escape is a major reason for tumor progression and metastasis. Some studies have suggested that a crosstalk may exist between glutamine metabolism and tumor immunology. GLUL was found to modulate macrophage skewing toward the M2 phenotype that was relevant for metastasis formation, where GLUL-deficient macrophages inhibited T Cell suppression, endothelial cell capillary formation as well cancer cell motility, and induced lymphocyte recruitment to prevent tumor metastasis (102). GLUL was also found to enhance HCC cell migration and invasion both *in vitro* and *in vivo*, and higher GLUL level independently predicted a poorer prognosis in HCC patients (103). In addition, Wu et al. suggested that glutamine metabolism could support highly immunosuppressive tumor-infiltrating immature myeloid cells with glutamine-derived α-ketoglutarate, and could also regulate their suppressive capacity through the glutamate-NMDA receptor axis, in which inhibiting GLS1 improved the efficacy of anti-PD-L1 treatment, with decreased Arginase1+ myeloid cells, increased CD8+, IFNγ+, as well as granzyme B+ T cells, and delayed tumor growth in an immunotherapy-resistant mouse model (104). Johnson et al. demonstrated that GLS1 plays an important role in T cell activation and subset specification (105). GLS1 could promote differentiation of Th17 cells but distinctly suppress differentiation and effector function of CD4 Th1 and CD8 CTL cells. Despite that chronic GLS deficiency could impair T cell responses, transient GLS inhibition by CB839 also showed enhanced Th1 and CD8 CTL effector function and long-lasting cell numbers *in vivo*, providing a novel hint that transient GLS inhibition may be used in combination with immunotherapy to enhance the treatment effect (105). JHU-083 is a new inhibitor synthesized by Jonathan D group which is the prodrug of glutamine antagonist DON (106). By concurrently using JHU-083, Leone RD et al. found that glutamine blockade enhanced the anti-tumor effects of the anti-PD-1 therapy compared with anti–PD-1 therapy alone. Glutamine blockade with JHU-083 monotherapy could also enhance endogenous antitumor immunity by triggering tumor immune rejection and adaptive immune memory without additional immunotherapy (106). Besides, targeting glutamine metabolism with JHU-083 inhibits both tumor growth and metastasis in an immune-dependent manner, including inhibiting infiltration of myeloid-derived suppressor cells, reprogramming myeloid-derived suppressor cells and tumor-associated macrophages from a suppressive to a proinflammatory phenotype, increasing immunogenic cell death and antigen presentation, and reducing kynurenine levels in both tumor and myeloid-derived cells by inhibiting IDO expression, which in turn inhibited the development of metastasis and further enhanced antitumor immunity (107). Given the low response rate as well as high tendency of adaptive or acquired resistance in cancer immunotherapy (108), investigating the relationship between glutamine metabolism and tumor immunology may provide an insightful treatment solution in the future.

Glutamine metabolism is also joined in the biologic interaction within the tumor microenvironment. Yang et al. found that cancer associated fibroblast (CAF) synthesize glutamine in glutamine-deficient tumor microenvironment to maintain glutamine-addicted ovarian cancer cell growth, where targeting glutamine synthetase in tumor stroma could reduce tumor weight and metastasis in orthotopic ovarian carcinoma mouse model, and the treatment effect can be further enhanced by co-targeting glutaminase in cancer cells (109). Due to the poor vascularization and hypoxic environment, PDAC tumors have been found to be commonly deprived of several nutrients, including glutamine (110). Pharmacologically, glutamine deprivation by glutamine analog DON leads to the induction of EMT through selectively up-regulating the EMT transcription factor Slug in both KPC mouse model and human PDAC cell lines, contributing to enhanced tumor migration and invasion capacities (111). Besides, under the hypoxic condition, Xiang et al. found that GLS1 was implicated in hypoxia-induced cancer cell invasion and metastasis, where GLS1 knockdown significantly suppressed CRC cell migration and invasion *in vitro*, as well as tumor growth and metastatic colonization *in vivo* (64). Besides, extracellular vesicles (EV) are wildly considered as an important bridge connecting cell communications in the tumor microenvironment and are involved in the process of pre-metastatic niche formation (112). In the LNCaP prostate cancer progression mode, Dorai et al. linked EV to glutamine metabolism, in which large EVs produced from highly bone metastatic C4-2B cells was significantly decreased when treated with glutaminase inhibitor BPTES, leading to an inhibition of bone metastasis in prostate cancer (113). Moreover, GLS1 inhibition combined with metformin treatment suppressed tumor growth and reduced metastatic progression in spontaneous metastasis mouse models with osteosarcoma (114).

Tumor metastasis is a natural and mostly inevitable process during the tumor progression, and also the leading cause of tumor-associated death. Given glutamine metabolism is involved in different phases of tumor metastasis development, genetically or pharmacologically targeting glutamine metabolism may suppress the initiation and progression of metastasis and provide a promising prospect in cancer treatment.

## Glutaminase Inhibitor Based Therapeutic Strategy

Due to the critical role of glutaminolysis in cancer metabolism, it has been a promising therapeutic target to combat cancers. As the first step of glutaminolysis, glutaminase convert glutamine to glutamate. This important role of glutaminase in glutamine metabolism makes it a valuable target for cancer therapy. The application of glutaminase inhibitors attenuates the glutamine to glutamate conversion, elevates intracellular ROS level and impairs antioxidant GSH production in cancer cells (15, 115, 116). Furthermore, the combination of glutaminase inhibitors with chemotherapy agents also increased sensitivity of cancer cells to chemotherapy in pancreatic cancer and ovarian cancer (59, 117, 118).

**Figure 3:**
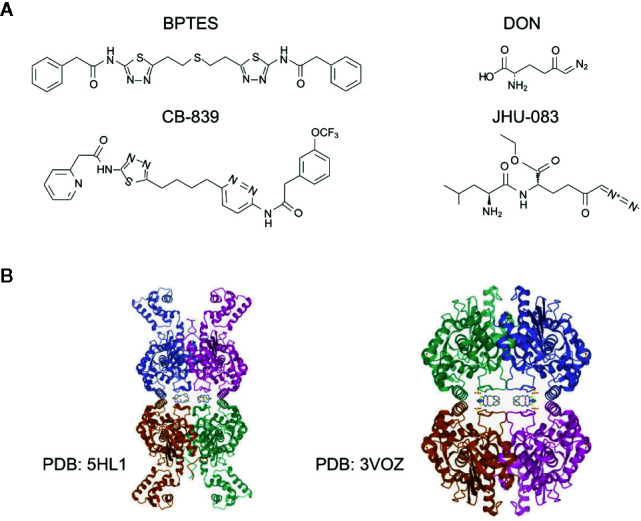
Structures of glutaminase inhibitors. **(A)** The structures of selected glutaminase inhibitors, including BPTES, CB-839, DON and JHU-083. **(B)** The structure and allosteric binding pocket of GLS1 (rcsb.org). Left, structure of GLS1 in complex with BPTES, PDB entry 3VOZ; right, structure of GLS1 in complex with CB-839, PDB entry 5HL1. The inhibitors are at the center of the structures.

To date, many potent small molecule inhibitors have been developed to target glutaminase, including DON, JHU-083, BPTES, CB-839, and compound 968 (119). DON is a glutamine antagonist, binds covalently to the enzyme active site and broadly inhibits glutamine-using enzymes, including glutaminase and glutamine amidotransferases involved in *de novo* nucleotide synthesis, amino acid synthesis, and hexosamine production (120). However, this ‘non-selective’ inhibition of glutamine metabolism induces high degree of toxicity, prevents its further investigation in glutamine targeting. To minimize the toxicity of DON, a prodrug strategy is developed (120). JHU-083 is a newly synthesized prodrug of DON, which can be administered in an inert state and then be activated preferentially in the tumor microenvironment through enzymatic cleavage, thus alleviating the previously reported toxicity of DON (106, 121). Other DON prodrugs such as Rais-5C and Nedelcovych-13d have also been reported (122–124). Unlike the glutamine mimetics, the allosteric inhibitors such as BPTES and CB-839, are selectively targeting glutaminase without disturbing other aspects of glutamine metabolism (25, 124). BPTES is now the most frequently used allosteric glutaminase inhibitor, which specifically inhibits kidney type glutaminase activity through the formation of an inactive complex (125). Though BPTES shows high specificity and efficiency in inhibiting cancer cell proliferation *in vitro*, the drawbacks of poor aqueous solubility and low bioavailability *in vivo* restrict its further applications in clinical trials (124). In order to improve drug solubility, several derivatives of BPTES were synthesized through structural modifications (119, 126–128). Later on, CB-839, a more potent, and orally bioavailable BPTES derivative was discovered. CB-839 shows a broad anti-proliferative activity in a number of cell lines in culture (42, 129, 130). Importantly, dozens of clinical trials of monotherapy or combination therapy with CB-839 are currently ongoing (42, 124). Another widely used glutaminase inhibitor is compound 968, a dibenzophenanthridine, which is first reported to be a GAC inhibitor and repressed oncogenic transformation in breast cancer cells, but is lately found by Lukey et al. to be a pan-glutaminase inhibitor with a moderate selectivity for GLS2 (65, 78). Recently, more potent GLS inhibitors were investigated, including CB-839 selenadiazole-derivatives CPD-20, CPD-23 (131), and Physapubescin I (132). Structures of selected inhibitors and the allosteric binding of GLS1 with BPTES and CB-839 are shown in **Figure 3** (66). However, less efforts have been made to target GLS2 due to its controversial roles in tumor suppression (26, 71, 92). Lee et al. reported a series of alkyl benzoquinones that preferentially inhibit GLS2 rather than GLS1, which function through the specific binding to an allosteric pocket at the C-terminal end of GLS2 monomer (133). Yeh et al. reported a class of thiazolidine-2,4-dione compounds targeting both GLS1 and GLS2, while moderately selective for GLS1 over GLS2 (134).

Despite the promising cell proliferation inhibition results observed *in vitro*, some cancer cells show resistance to glutaminase inhibitors. More importantly, the *in vivo* data of glutaminase inhibition is still quite limited and shows controversial results (42, 130, 135). Gross et al. reported significant antitumor activities of CB-839 in two xenograft models, a patient-derived TNBC model and a basal like HER2+ cell line model (JIMT-1) (42). Lee et al. reported a successful inhibition of undifferentiated pleomorphic sarcoma (UPS) tumor growth with CB-839 (135). Combination therapy of CB-839 and PARP inhibitor olaparib also showed prolonged survival in a xenograft model of ovarian cancer (136). However, Biancur et al. found no antitumor effect of CB-839 in both autochthonous and subcutaneous mouse models of PDAC (130). Their work suggested that compensatory metabolic networks emerged during glutaminase inhibition, with the activation of alternative pathways of glutamate production. Nevertheless, the high clearance rate of CB-839 in mice should also be considered (42). Noteworthy, reducing cell culture medium nutrients to physiological levels also compromised the sensitivity of lung cancer cells to glutaminase inhibitors (137). Singleton et al. found that CB-839 activity was significantly compromised in three dimensional spheroids assay compared with two dimensional monolayer culture in TNBC cells (138). Davidson et al. reported that KRAS-driven lung tumors require pyruvate carboxylase and pyruvate dehydrogenase, and are less dependent on glutaminase than cultured cells (139), suggesting a crucial impact of tumor microenvironment in glutamine metabolism and glutaminase inhibition. In addition, Muir et al. showed that cystine levels dictate glutamine dependence *via* the cystine/glutamate antiporter SLC7A11 (xCT) and concurrent high expression of GLS and xCT may predict response to glutaminase inhibition (78, 137, 140). Grinde et al. found that addiction to proline synthesis from glutamine is associated with response to CB-839 in breast cancer (141).

**Figure 4:**
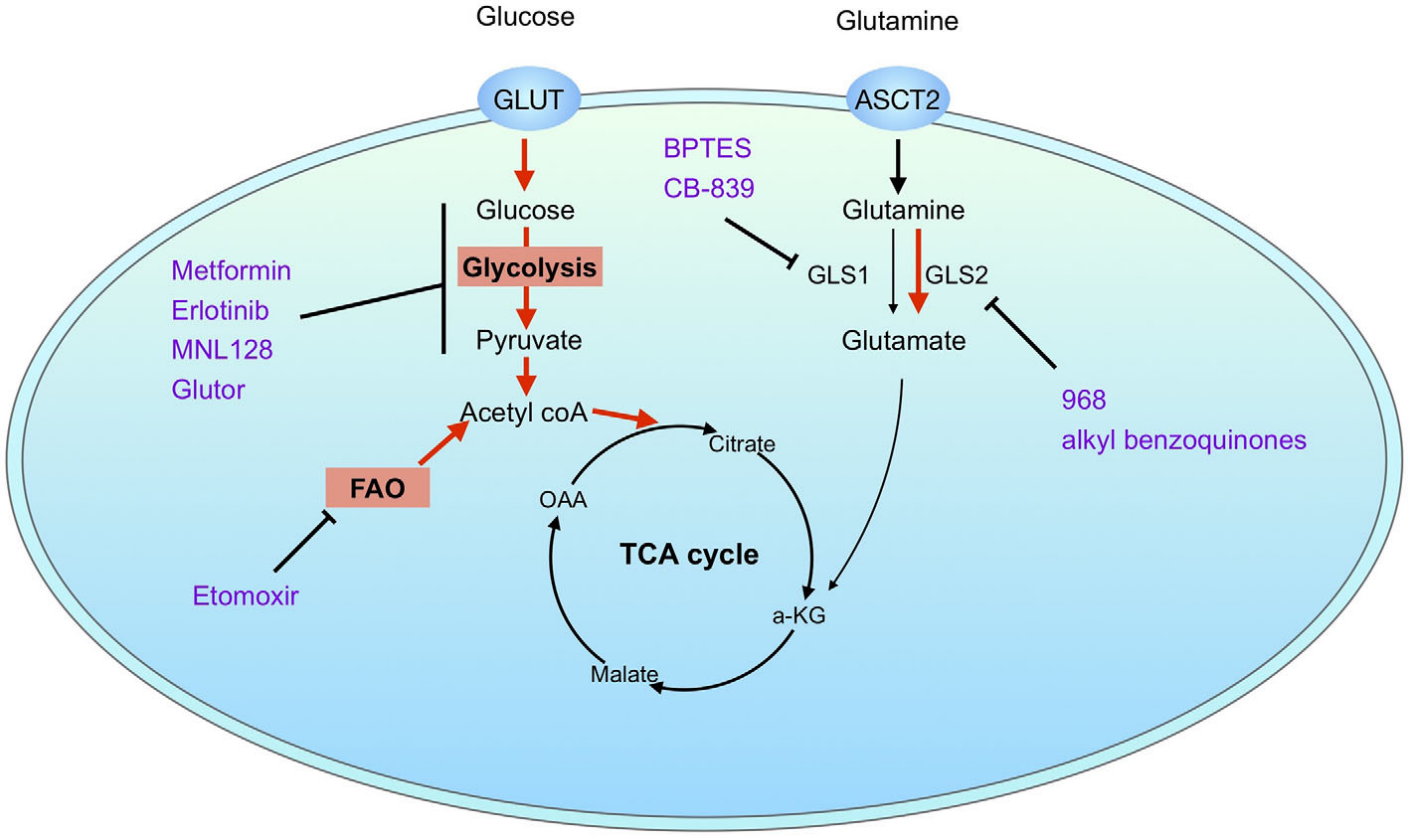
Mechanisms of glutaminase inhibition resistance. Resistance to pharmacological glutaminase inhibition may be explained by differential expression of glutaminase isoenzymes or activation of alternative metabolic pathways. Combination therapy with inhibitors targeting alternative metabolic pathways, such as glycolysis and fatty acid oxidation, helps to overcome glutaminase inhibition resistance. Possible mechanisms of glutaminase inhibition resistance are marked in red. Inhibitors are marked in purple.

The questions then arise: what is the molecular mechanism of glutaminase inhibition resistance and how could we overcome the therapy resisatnce? Firstly, as the most frequently used glutaminase inhibitors such as BPTES and CB-839 are GLS1 selective, the resistance to glutaminase inhibition may be due to the differential expression of GLS1 and GLS2 in cells, as demonstrated in luminal and basal-like breast cancer cells (78). Application of a pan-glutaminase inhibitor 968 suppresses BPTES-resistant breast cancer growth. Importantly, a number of studies have demonstrated that glutaminase inhibition could be rescued by alternative metabolic pathways, such as glycolysis and fatty acid oxidation (FAO) (130, 138, 142). A combinatorial strategy may help to overcome glutaminase inhibition resistance. Several inhibitors targeting glycolysis have demonstrated a synergistic effect with glutaminase inhibitor, such as metformin (115, 143, 144), Erlotinib (EGFR inhibitor) (145), MLN128 (mTOR inhibitor) (52), and Glutor (glucose uptake inhibitor) (146). Co-inhibition of FAO with etomoxir (CPT1 inhibitor) as well inhibits the cell proliferation in resistant cells (130, 142). However, the combination of CB-839 and etomoxir was lethal in mouse models. In addition, combined therapy targeting oxidative stress response also show enhancement of the sensitivity to glutaminase inhibition (60, 130). Together, combinatorial strategies show the effectiveness in overcoming the glutaminase inhibition resistance. A summarized diagram of glutaminase inhibition resistance is showed in **Figure 4**.

Although as a promising therapeutic approach to combat cancer, limited clinical research data of glutaminase inhibition is available. In the last few years, CB-839 is the only glutaminase inhibitor undergoing clinical trials. Most recently, a new inhibitor DRP-104 (glutamine antagonist) is now entering clinical trials (NCT04471415). However, most of the trials are in a stage of phase I/II, evaluating the safety and tolerability of the inhibitors. Nevertheless, results of CANTATA (NCT03428217) showed encouraging clinical activity and tolerability of combination therapy of CB-839 plus cabozantinib in metastatic renal cell cancer (147). Supportively, Zhao et al. reported that combination of CB-839 and 5-fluorouracil induced PIK3CA-mutant tumor regression in CRC xenograft models (148). Importantly, an exploratory analysis of a phase I clinical trial (NCT02861300) showed a trend of better response to combination therapy of CB-839 plus capecitabine (prodrug of 5-fluorouracil) in PIK3CA-mutant CRC patients as compared to PIK3CA-WT cohort (148). More data are needed to evaluate the efficiency of glutaminase inhibition in clinical scenarios.

## Conclusions

Uncontrolled cell growth is an essential feature of cancers, which is supported by the augmented glycolysis as well as glutaminolysis. Studies of cancer metabolic reprogramming provide new insights into the nature of malignancy and reveal a potent target to combat cancer. Despite the pivotal role of glucose, the importance of glutamine metabolism in cancer is well recognized. In this review, we updated the current understanding of glutaminolysis in cancer from the view of glutaminase isoenzymes and summarized the glutaminase inhibitor based therapeutic strategies. However, high metabolic heterogeneity increases the complexity of metabolic targeting therapies. Pharmacological inhibition of glutaminases gives different responses in various cancers, which may be due to the differential expression of glutaminase isoenzymes or emerge of alternative metabolic pathways. Combinatorial strategies have shown promising synergistic effects in some context and may help overcome glutaminase inhibition resistance. Identification of glutaminase inhibitor sensitive cancers and optimization of combination therapies would be an interesting focus for targeting glutaminolysis in a variety of cancers.

## Author Contributions

All authors contributed to the article and approved the submitted version.

## Funding

This work was supported by Köln Fortune Program/Faculty of Medicine to YZ, University of Cologne (ID: 2680154501).

## Conflict of Interest

The authors declare that the research was conducted in the absence of any commercial or financial relationships that could be construed as a potential conflict of interest.

## Abbreviations

CAF, cancer associated fibroblast; CRC, colorectal cancer; DON, 6-diazo-5-oxo-L-norleucine; EMT, epithelial-mesenchymal transition; EV, extracellular vesicles; FAO, fatty acid oxidation; GAB, glutaminase B; GAC, glutaminase C; GLS, glutaminase; GLUD, glutamate dehydrogenase; GLUL, glutamine synthetase; GOT, glutamic-oxaloacetic transaminase; GPT2, glutamate pyruvate transaminase 2; GSH, glutathione; HCC, hepatocellular carcinoma; HIF, hypoxia-inducible factor; ICC, intrahepatic cholangiocarcinoma; IDH, isocitrate dehydrogenases; KEAP1, Kelch-like ECH-associated protein 1; KGA, kidney-type glutaminase; LKB1, liver kinase B1; lncRNA, long non-coding RNA; NEAA, non-essential amino acid; NFR2, Nuclear factor erythroid 2-related factor 2; PDAC, pancreatic ductal adenocarcinoma; PTM, post-translational modification; ROS, reactive oxygen species; TCA cycle, citric acid cycle; TNBC, triple-negative breast cancer; UPS, pleomorphic sarcoma; xCT, cystine/glutamate antiporter; α-KG, α-ketoglutarate.
